# The Diagnostic Role of Serum Inflammatory and Soluble Proteins on Dementia Subtypes: Correlation with Cognitive and Functional Decline

**DOI:** 10.1155/2007/432190

**Published:** 2008-03-05

**Authors:** Candan Öztürk, Aynur Özge, Osman Özgür Yalın, &Idot;. Arda Yılmaz, Nuran Delialioglu, Çilem Yıldız, Bahar Tesdelen, Cigdem Kudiaki

**Affiliations:** Mersin University School of Medicine and Mersin University School of Science and LettersMersinTurkey

**Keywords:** Alzheimer’s disease (AD), vascular dementia (VD), mild cognitive impairment (MCI), cognitive decline, functional decline, inflammatory, cytokine, soluble protein

## Abstract

In the past years, the possible involvement of inflammation in the pathogenesis of dementia has been the subject of several investigations. However there are restricted data about the profile of the inflammatory and soluble proteins in well evaluated Alzheimer’s disease (AD), vascular dementia (VD), mild cognitive impairment (MCI) and healthy controls. There are also no reliable data regarding the relationship between the overlapping protein levels and cognitive or functional decline. We measured levels of IL-1*β*, IL-2, IL-6, IL-18, TNF-*α*, *β*-Amlyloid 1–40 and *α*_1_-antichymotrypsin levels in plasma in groups of total 82 subjects with AD, MCI, VD and controls using enzyme-linked immunosorbent assay (ELISA) method. Our study samples showed high levels of proinflammatory cytokine levels (especially IL-18) in all patient groups but only high levels of *α*_1_-antichymotrypsine in VD patients compared to controls. There is no significant correlation between the laboratory and clinical variables except for a link between IL-1*β* and NPI scores of AD. In conclusion, this study yielded evidence of some shared mechanisms underlying AD and VD and thus motivates further studies of inflammatory markers in various types of dementia and MCI.

## 1. Introduction

Alzheimer's disease (AD) is the major form of dementia, affecting roughly 1% of people at the age of 65 and approximately doubling in incidence for every additional 5 years of age [39]. No more than 85% of clinically diagnosed AD could be confirmed by autopsy [17]. Thus, identification of diagnostic biomarkers in the plasma that would allow earlier and more accurate diagnosis of AD and differentiation from other common dementia types is an emerging goal of research [24].

The possible involvement of inflammation in the pathogenesis of dementia has been the subject of several investigations. Even though the inflammatory mechanisms involved in the pathogenetic cascade are currently unknown the relevance of inflammation in the pathogenesis of these conditions is a still debated. Immunohistochemical, biochemical, and molecular studies indicate that a chronic inflammatory reaction is present in selected brain areas of AD patients [5]. In particular, the upregulation of cytokines [21], chemokines [73], acute phase proteins [29], activated complement factors [69], and free radicals [63] has been clearly established in individuals with AD. Overproduced by activat-

ISSN 0953-4180/07/$17.00 © 2007 – IOS Press and the authors. All rights reserved ed microglia [16] and reactive astrocytes [44], these mediators have been reported to be found in the vicinity of $\beta$-amyloid deposits and neurofibrillary tangles and may play a role in the pathogenesis of the AD and partially vascular dementia (VD) subjects [42]. There is increasing evidence that VD shares common elements with AD especially in terms of neuroinflammation based data. This hypothesis is supported by evidence that AD and VD share many similar risk factors, overlapping clinical symptoms and cerebro-microvascular pathology [76]. On the other hand, genetic studies indicate that the risk of AD is substantially influenced by a total of 10 polymorphisms in the inflammatory molecules, namely, interleukin (IL)-1$\beta$, IL-1$\alpha$, IL-6, tumor necrosis factor (TNF)-$\alpha$, $\alpha$2-macroglobulin, and $\alpha$1-antichimotripsin, an acute phase protein [26,38,50]. Phenotypic consequences of these genetic alterations for each of these molecules are currently under investigation.

Pro-inflammatory cytokines could affect A$\beta$ formation by raising the susceptibility for A$\beta$ deposition or aggregation, transcriptionally upregulate b-secretase (BACE1) mRNA, protein and enzymatic activity. The generaly accepted amyloid hypothesis states that formation of amyloid peptides (A$\beta$) by neurons is the primary trigger of the pathogenesis of AD, and A$\beta$ deposition was considered to be the cause of the disease within the last few years. Evidence supporting the amyloid hypothesis has been recently reviewed [69]. However, it is still unclear how A$\beta$ causes cell damage. Several mechanisms have been proposed. One view suggests that A$\beta$ protofibrils activate microglia, inciting an inflammatory response and release of neurotoxins or neurotoxic cytokines [59]. It has been speculated that this inflammatory response associated with the presence of neuritic plaques is secondary to A$\beta$ accumulation and could be involved in neuronal damage and with the progression of the disease. Activated microglia and reactive astrocytes surrounding extracellular deposits of amyloid $\beta$-protein initiate an inflammatory response characterised by a local cytokine-mediated acute phase response, activation of the complement cascade and subsequent further cell damage [64]. There are also no reliable data concerning the determination of A$\beta$40 in the peripheral circulation as a marker of particular dementia subtypes. No other supportive data are available regarding the correlation between A$\beta$40 and cytokine levels and the severity of the disease.

A number of studies have addressed the issue of peripheral inflammatory molecules, including cytokines, as possible markers of AD and other dementia types, and they analyzed whether the presence of these molecules in blood could be related to the severity or the progression of the disease [21,26,29,38,50,73]. Unfortunately, available data are often contradictory and do not allow us to draw definite conclusions. Applying accurate diagnostic procedures and rigorous exclusion criteria, there is no reliable data regarding the presence of a panel of pro- and anti-inflammatory cytokines in different types of dementia including AD, VD, MCI, and in age matched subjects with no cognitive impairment.

We performed this cross-sectional case control study in order to determine the frequency of some candidate cytokine, acute phase protein and A$\beta$-40 abnormalities in the main dementia subgroups (MCI, AD, VD) and to clarify both the importance of inflammatory markers for dementia diagnosis and correlation between clinical and laboratory variables.

## 2. Patients and method

Patients with diagnosis of possible AD, MCI and VD were selected at the Mersin University, Faculty of Medicine, Neurology Outpatient Department, Dementia Subunits. The investigation was carried out in accordance with the Declaration of Helsinki. Informed written consent to participate in the study was obtained from the subjects or, when necessary, from their legal guardians under local institutional review board supervision and approval. The study protocol was approved by the local ethical committee of Mersin University, Faculty of Medicine also.

The study groups consisted of patients as follows; 29 patients (9 males and 20 females, age 77.7 $\pm$ 8.3 years) were diagnosed as probable AD according to the criteria of NINCDS-ADRDA [41], 17 patients (10 males and 7 females, age 71.8 $\pm$ 11.5 years) were diagnosed as probable VD according to the criteria of NINDS-AIREN [56] and 13 patients (7 males and 6 females, age 68.0 $\pm$ 6.7 years) were diagnosed as probable MCI according to the criteria of American Academy of Neurology [53]. Patients with Hachinski ischemic score (modified according Loeb & Gandolfo [33]) greater than 5 were assigned to the VD group. The control group consisted of 23 subjects (11 males and 12 females, age 64.7 $\pm$ 8.5 years) who had no cognitive decline and consisted of the spouses of the patients and the relatives of the hospital personnel.

### 2.1. Neuropsychological evaluation included the following measurements

The areas of neuropsychological functioning examined by the educated physician under supervision of neuropsychologist and specific tests administered were as follows:

1. Screening for cognitive impairment; Standardized Mini Mental State Examination (MMSE) [43],
2. Screening for dementia subtype; Hachinski Ischemic Score (HIS) [33]
3. Activities of daily living and functional capacity; Physical self- maintenance scale (PSMS), Instrumental activities of daily living scale (IADL), Modified activities of daily living scale (MADL) [28],
4. Screening for neuropsychological impairment; Neuropsychological Inventory (NPI) [12],
5. Staging of impairment; Global deterioration scale (GDS) [54] and Clinical dementia rating (CDR) [22].

A comprehensive somatic, psychiatric, and sociodemographic history was taken from all subjects who then underwent thorough psychiatric and neurological examination, EEG, and neuroimaging (CT or MRI). Total blood count and blood chemistry including C reactive protein, thyroid function, vitamin B12, folic acid were evaluated. Patients with a psychiatric, neurological, inflammatory or infectious disease or with a history of immunological or malignant disease were excluded, as well as persons with abnormal white blood cell count, C reactive protein or signs of malnutrition. Subjects who reported any major vascular events (myocardial infarction, cerebrovascular diseases, etc) were also excluded from the study. Further exclusion criteria were the intake of immunologically relevant or psychotropic drugs.

### 2.2. Sample collection and procedure

Blood samples (10 ml) were collected in tubes containing EDTA. They were centrifuged at 3000 rpm for 10 minutes, and sera were stored at $-80^\circ$C until the end of the specimen collection time.

IL-1$\beta$, IL-6, IL-18 and TNF-$\alpha$ were studied for proinflammatory cytokines. IL-2 was studied for anti-inflammatory cytokines. $\beta$-Amlyloid 1–40 was studied for soluble protein. Lastly CRP and $\alpha_1$-antichymotrypsin were studied for acute phase proteins. IL-1$\beta$, IL-2, IL-6, IL-18, TNF-$\alpha$, $\beta$-Amlyloid 1-40 and $\alpha_1$-antichymotrypsin levels were determinated in sera samples using commercially available ELISA kits according to manufacturer's instructions (064602 for IL-1$\beta$, 063901 for IL-2, 063202 for IL-6, 042 for IL-18, 064608 for TNF-$\alpha$, Biosoorce International, Nivelles/Belgium by ELISA and 5082210A for $\beta$-Amlyloid 1-40; Biotek EL800/USA; K6750-061117 for $\alpha_1$-antichymotrypsin by Immun Diagnostic/Benheim/ Germany Biotek EL800/USA). There was no cross-reactivity of monoclonal antibodies for other cytokines. The optical density of each well was measured at 490 nm by an Immuno Reader U 2000. The detection limits were as follows: IL-1$\beta$ $<$ 1 pg/ml, IL-2 $<$ 4 pg/ml, IL-6 $<$ 2 pg/ml, IL-18 $<$ 12.5 pg/ml and TNF-$\alpha$ $<$1.7 pg/ml, A$\beta$-40 $<$6 pg/ml, and $\alpha_1$-anticymotrypsin $<$98.22 pg/ml. All values below the detection levels were considered to be negative. High sensitive CRP levels were determined in sera samples using commercially available nephalometry kits according to manufacturer's instructions (number of 167545A by nephalometric systems; Dade Behring BN100/ Marburg/ Germany). Patients and control subjects with C-reactive protein concentration $>$ 20 $\mu$g/ml were excluded from the study (Normal ranges of CRP is between 0 to 5 mg/L in our laboratory database). At the end of the study, the following abnormality ranges were calculated according to determined calc OD values; IL-1$\beta$ $>$28,662 pg/ml, IL-6$>$43,678 pg/ml, IL-18$>$ 140.6 pg/ml, TNF-$\alpha$ $>$ 67,041 pg/ml and A$\beta$-40$>$ 1.729 pg/ml. IL-2 did not show any abnormal result in our study sample.

### 2.3. Statistical analysis

Differences in demographic variables were determined with a Student's $t$-test. Chi-square contingency test was used for comparison of frequency distributions between the groups. Serum data were expressed as mean $\pm$ SD. Normality of distributions was examined using a Kolmogorov-Smirnov test. Log-transformation did not improve normality for the serum markers. Therefore, all analyses on serum variables were performed with nonparametric tests. Differences between the serum concentrations of all neurological patients together and controls were determined using the Mann–Whitney $U$-test. Differences between groups were examined using ANOVA after calculation of the ranks of the data, with Scheffé's test as post hoc analysis. The relationships were analyzed by the methods of regression and correlation analysis as appropriate.

Table 1
Demographic and neuropsychological features of the study groups

| | AD group ($n = 29$) | MCI group ($n = 13$) | VD group ($n = 17$) | Controls ($n = 23$) |
|---|---|---|---|---|
| Age | 77.7 ± 8.3 | 68.0 ± 6.7 | 71.8 ± 11.5 | 64.7 ± 8.5 |
| Education (year) | 5.4 ± 4.9 | 9.4 ± 4.3 | 5.2 ± 4.0 | 4.7 ± 4.0 |
| Gender F/M | 20 (69%)/ 9(31%) | 6(46.2%)/ 7(53.8%) | 7(41.2%)/ 10 (58.8%) | 12 (52.1%)/ 11 (47.8%) |
| MMSE-total | 10.9 ± 7.0 | 26.3 ± 1.6 | 14.9 ± 7.5 | |
| HIS | 2.6 ± 1.9 | 2.6 ± 2.5 | 10.1 ± 2.9 | |
| PSMS | 19.7 ± 4.8 | 6.6 ± 1.8 | 15.9 ± 7.4 | |
| IADL | 7.1 ± 3.9 | 0.3 ± 0.6 | 4.8 ± 4.9 | |
| MADL | 11.1 ± 3.1 | 1.0 ± 1.4 | 9.9 ± 4.9 | |
| NPI total score | 16.8 ± 14.7 | 2.9 ± 4.1 | 12.4 ± 13.2 | |
| CDR score; n(%) | | | | |
| 0.5 | 3 (10.3) | 13 (100) | 5 (29.4) | |
| 1 | 2 (6.9) | | 4 (23.5) | |
| 2 | 16 (55.2) | | 5 (29.4) | |
| 3 | 8 (27.6) | | 3 (17.6) | |
| GDS score; n(%) | | | | |
| Stage 2 | – | 12 (92.3) | – | |
| Stage 3 | 4 (13.8) | 1 (7.7) | 6 (35.2) | |
| Stage 4 | 6 (20.7) | | 2 (11.8) | |
| Stage 5 | 9 (31.0) | | 7 (41.2) | |
| Stage 6 | 5 (17.2) | | 1 (5.9) | |
| Stage 7 | 5 (17.2) | | 1 (5.9) | |

Logistic regression was performed with AD against other groups. The variables were entered with a forward stepwise mode based on significant improvement in Log Likelihood Ratios and significance of the included variables, with age included in every model. Results with significance level of $p < 0.05$ are reported.

## 3. Results

A total of 59 patients and 23 healthy subjects were included in this study. The patient groups were composed of 29 (49.2%) patients with AD, 13 patients (22%) with MCI and 17 patients (28.8%) with VD. There were no significant age and sex difference between the groups. Descriptive data of the subjects are given in Table 1.

All cognitive and functional scores of MCI group were better than AD and VD patients. On the other hand, except for the PSMS score, all cognitive and functional scores of the AD and VD subjects did not show any significant difference. In terms of severity of general dementia, these subjects disclosed similarities. Additionally cognitive decline was generally correlated with functional decline independent from dementia type.

Excluding the positive correlation between IL-6 levels and total NPI scores of the AD patients, there was no significant correlation between the cognitive or functional scores and laboratory data. Only AD patients showed a significant correlation between TNF-$\alpha$ and IL-1$\beta$ (CC: 0.68, $p = 0.000$) and IL-6 (CC: 0.59, $p = 0.001$) abnormalities (Please refer to Table 2 for details).

Binary logistic regression analysis using Forward LR selection method showed only MMSE total scores had important association with IL-1$\beta$ levels (Exp-B: −2.978, $p = 0.043$) in AD when age, GDS score and IADL were covariates. However these variables were not associated with other cytokine or protein levels in this model.

## 4. Discussion

In this study in order to examine the inflammatory markers of the main dementia subtypes and to determine a possible distinctive marker we measured the concentrations of cytokines IL-1$\beta$, IL-2, IL-6, IL-18 and TNF-$\alpha$, acute phase proteins ($\alpha_1$-antichymotrypsine) and as well as of the soluble proteins A$\beta$-40 in serum of AD, MCI, VD patients and matched controls.

During the last decade, it has been established that there is a local chronic inflammatory process in the AD brain. Amyloid deposition in the AD brain elicits a range of reactive inflammatory responses including astrocytosis, microgliosis, upregulation of proinflammatory cytokines, complement activation, and acute phase

Table 2
Laboratory data of the study subjects

| Parameters | AD group ($n = 29$) | MCI group ($n = 13$) | VD group ($n = 17$) | Controls ($n = 23$) |
|---|---|---|---|---|
| IL-1$\beta$ | | | | |
| n (%) | 4 (13.7) | 1 (7.7) | – | – |
| Mean $\pm$ SD | 43.5 $\pm$ 17.7 | 5.12 | – | – |
| 95% CI (low-up) | 15.3–71.7 | – | – | – |
| IL-6 | | | | |
| n (%) | 5 (17.2) | 1 (7.7) | – | 1 (4.3) |
| Mean $\pm$ SD | 91.7 $\pm$ 41.0 | 364.9 | – | 310.8 |
| 95% CI (low-up) | 197.8–425.8 | – | – | – |
| IL-18 | | | | |
| n (%) | 7 (24.1) | 1 (7.7) | 4 (23.5) | 1 (4.3) |
| Mean $\pm$ SD | 211.5 $\pm$ 93.4 | 230.9 | 218.6 $\pm$ 76.4 | 330.4 |
| 95% CI (low-up) | 125.0–298.0 | – | 97.0–340.2 | – |
| TNF-$\alpha$ | | | | |
| n (%) | 2 (6.8) | – | – | – |
| Mean $\pm$ SD | | – | – | – |
| 95% CI (low-up) | | – | – | – |
| A$\beta$-40 | | | | |
| n (%) | – | 1 (7.7) | 1 (5.8) | – |
| Mean $\pm$ SD | – | 5.5 | 3.1 | – |
| 95% CI (low-up) | – | – | – | – |
| $\alpha_1-$anitrypsine | | | | |
| n (%) | – | 2 (15.3) | 6 (35.2)** | 1 (4.3) |
| Mean $\pm$ SD | – | 157.1 $\pm$ 50.4 | 127.6 $\pm$ 24.8 | 103.9 |
| 95% CI (low-up) | – | $-$295.7 $\pm$ 609.9 | 101.5–153.6 | – |

Note: IL-2 levels did not show any significant abnormality.
*$p < 0.05$, **$p < 0.001$, SD: Standard deviation (by chi-square test).

reactions [66]. Activated microglia and inflammatory mediators are found in close association with or within the amyloid plaques, indicating important interactions [32]. In another aspect, there is some suggestive data about a general decline of immune responsiveness in AD [55].

This study focused on the following questions:

– What is the role of serum cytokine abnormalities in the diagnosis of the main dementia subtypes?
– Is there any correlation between serum cytokine and protein levels of these subjects?
– Is there any correlation between these markers and cognitive, psycholgical or functional decline of the subjects?

Reports on peripheral cytokine secretion in some dementia types are inconsistent. Obvious methodological differences among studies, including inclusion criteria and technical variations contribute to the great variability of data. Sample sizes show considerable differences, and patient groups differ with respect to stage of dementia, further pathological conditions and drug intake. Moreover, varying cytokine levels may also be due to genetic polymorphisms [7]. Therefore, the measurement of a single cytokine does not allow us to reach to any conclusions on disease dependent effects. Rather, an overlapping set of cytokines, as presented in this study, combined with soluble protein levels and acute phase proteins in three main dementia subtypes may provide more information. Most importantly, cytokine production is highly dependent on health status. Previously reported higher levels of pro-inflammatory cytokines in aged persons as well as in AD may reflect an underlying but undiagnosed disease state [8]. To overcome this probability we excluded each person with the slightest sign of infection or another medical disease in our study, because any comorbidity could influence the cytokine production.

Indicators of brain inflammation, increased [5,7,9, 45,72], unchanged [27,60] and decreased [58] levels of proinflammatory cytokines in CSF have been found in AD. As expected, there are similar inconsistencies for the findings in serum: Some working groups reported elevated levels of proinflammatory cytokines [25, 31,58]; others did not see any changes [18,27]; while some found a decrease of proinflammatory cytokine secretion [11,13,49,57]. Results regarding cytokines in CSF and sera have also conflicted [23,63]. These discrepancies have mostly been attributed to technically different approaches and to different criteria to choose patient groups as well as control groups. Moreover, most of the studies report very low cytokine levels close to their detection limit, so that statistical evaluation is restricted [55]. At this point, none of them reported the diagnostic importance of the cytokines on the dementia subgroups. Our study results showed a slight increase that was statistically significant, in the levels of the proinflammatory cytokines in all patient groups compared to controls. The most important proinflammatory cytokine abnormality has been determined in IL-18. Also antiinflammatory cytokines did not show any abnormal levels.

Although it has been stated that the neuroinflammatory events found in the brain and CSF of AD patients seem to be limited to the CNS without direct association of a peripheral inflammation [9], McGeer EG [39] et al. proposed that more research for defining the precise roles of chemokines in neuroinflammation in general and AD in particular is a priority for the future. As a rarely studied cytokine in AD patients, IL-18 (previously known as interferon-$\gamma$ inducing factor) is a pro-inflammatory cytokine that has been demonstrated in the normal human CNS and found to be increased in closed head injuries [48,75]. A recent study's [32] results suggested that the serum levels of IL-18 did not differ between the control subjects and the patient groups, or between MCI and severe AD. This suggests a normal feed-back regulation of inflammatory and anti-inflammatory reactions, and argues against a role of IL-18 in AD pathology, further supported by the lack of correlation between IL-18 and MMSE scores. However, our study results did not supported an important correlation between IL-18 in any groups of the patients; but IL-6 levels were correlated with higher NPI scores, commonly expected in high grades of the dementia. A possible cause of the lack of any correlation between cytokine levels and severity of the disease can be atrributed to small study samples. On the other hand, logistic regression analysis supported an important association betweeen MMSE scores and IL-1$\beta$ levels in AD patients. These associations suggested a possible relationship between low MMSE scores and high NPI scores in dementia. In this aspect more comprehensive data are also required in order to say which cytokine is directly correlated with which NPI and MMSE scores.

At this point there are restricted data about the profile of overlapping cytokine levels on the other causes of the cognitive impairment such as MCI as a potential cause of the future AD. As an important data, Tarkowski et al. [62] suggested that increased production of the proinflammatory cytokine, TNF-$\alpha$, and decreased production of the anti-inflammatory cytokine TGF-$\beta$ in patients with MCI constitutes a risk to develop AD. Our small sample demonstrated cytokine profiles of MCI located between AD and healthy controls, supporting the mentioned study.

Several recent studies have provided insight into the possible role of inflammatory processes in the development of brain ischemia and multi-infarct cognitive impairment, as demonstrated by the accumulation of inflammatory cells and mediators in the ischemic brain [4, 14,30,61,67]. Vila et al. [66] reported that interleukin-6 (IL-6) participates in the acute phase response that follows cerebral ischemia and that an association exists between high levels of IL-6 and early neurological worsening. An important clinically based cross-sectional study suggested that the inflammatory activations in the CNS might be associated with some part of VD patients, and measurement of CSF IL-6 might provide a clue to differential diagnosis of dementia [67]. Our study samples showed that VD patients showed only IL-18 abnormality and the frequency was lower than in AD but higher than in MCI and in controls. These levels did not show any correlation with cogntive or functional scores, possibly because of the small sample sizes.

High plasma IL-6 levels in healthy aged subjects were related to greater cognitive decline in a cross-sectional study [70]. Also, an important study [57] showed that peripheral IL-1beta, IL-6 and TNF-$\alpha$ secretion were negatively correlated with the severity of dementia, quantified by the MMSE. However, our data showed no important correlation between the cytokine levels and severity of evaluated by both the CDR and the GDS in all of the patient groups, except for the NPI scores.

IL-1 and IL-6 are able to induce human astrocytes to over-express the gene for the acute phase protein $\alpha$1-antichymotrypsin (ACT) [47]. ACT mRNA was also shown to be expressed at higher levels by astrocytes in the gray matter of AD brains as compared to controls [51] and the ACT was shown to be tightly associated with amyloid plaques in AD brain [1,2]. The serum concentrations of the inflammatory markers interleukin-6 (IL-6) and $\alpha_1$-antichymotrypsin were increased in AD patients compared to controls, though these findings have not been replicated by all other studies [6,10,31]. There are restricted data about the status of the ACT in MCI or VD. Our samples showed a high ratio of ACT levels in VD when compared to MCI and healthy controls (for details please refer to Table 2). These data supported a commonly discussed shared mechanism of AD and VD and the potential role of ACT in VD pathogenesis.

The $\beta$-amyloid deposition that constitutes the plaques is composed of a 39–42 amino acid peptide (A$\beta$), which is the proteolytic product of the amyloid precursor protein (APP) [34]. CSF A$\beta$ levels do not correlate with plasma A$\beta$ levels in individual patients [20, 41,65]. There is some evidence that brain-to-plasma A$\beta$ efflux could be a peripheral indicator of the extent of cerebral amyloid deposition, even before onset of AD symptoms [15]. A$\beta$ can cause peripherally circulating macrophages to cross the blood–brain barrier via chemokine recruitment, possibly leading to an increased inflammatory burden [19]. Although not useful in diagnostic terms, plasma A$\beta$ measures can also be evaluated in the context of AD prediction, progression, and therapeutic monitoring [35]. In cross-sectional studies, though, plasma A$\beta$ levels did not correlate with measures of progression or dementia severity [20,41]. In our study sample, we could only measure A$\beta$-40. In our study the results of A$\beta$-40 showed no abnormality in the peripheral sera samples. There was also no important association between A$\beta$-40 and other inflammatory markers, which might be due to low sample size also.

This study is limited due to the small number of subgroup subjects and the heterogeneity of patients in terms of age, disease duration and severity. Also the unavoidable differences of the subjects' age group should be kept in mind, although the regression analaysis we did not show any effect of age on cytokine abnormalities. However, the present data support alternative views on the hypothesis of a mere combination of inflammation-mediated pathogenesis and similar clues of neurodegeneration of the AD, MCI and VD.

As a conclusion, the study provided data supporting some shared mechanisms of AD and VD and motivates the development of more extensive studies of dementia and MCI.

## Acknowledgements

This study was supported by the Mersin University Scientific Research Support Foundation (365 BAP-TFTTB 2006-3).
